# Local Ablative Treatment Improves Survival in ESCC Patients With Specific Metastases, 2010–2016: A Population-Based SEER Analysis

**DOI:** 10.3389/fonc.2022.783752

**Published:** 2022-06-16

**Authors:** Hui Yang, Kunlun Wang, Yan Li, Shenglei Li, Ling Yuan, Hong Ge

**Affiliations:** The Affiliated Cancer Hospital of Zhengzhou University & Henan Cancer Hospital, Zhengzhou, China

**Keywords:** esophageal squamous cell cancer, local ablative treatment, chemotherapy, metastases, radiotherapy, surgery, prognosis, SEER

## Abstract

**Background:**

We aimed to explore the role of local ablative treatment (LAT) in metastatic esophageal squamous cell cancer (ESCC) patients who received chemotherapy and identify patients who will most likely benefit.

**Methods:**

We analyzed data of metastatic ESCC patients from the Surveillance, Epidemiology, and End Results (SEER) database between 2010 and 2016. The chi-square test was used to evaluate the unadjusted clinicopathological categorical variables between the two groups. Univariate and multivariate Cox regression analyses were conducted to identify independent prognostic factors of overall survival. Propensity score matching (PSM) was used to adjust the differences between the two groups.

**Results:**

Overall, 720 metastatic ESCC patients treated with chemotherapy were analyzed in this study; 63.2% of patients (*n* = 455) received LAT, including radiotherapy (*n* = 444), primary site surgery (*n* = 12), or lymph node dissection (*n* = 27). Gender (HR = 1.220, 95% CI: 1.024–1.453, *p* = 0.026), bone metastases (HR = 1.559, 95% CI: 1.292–1.882, *p* < 0.001), and liver metastases (HR = 1.457, 95% CI: 1.237–1.716, *p* < 0.001) were independent prognostic factors in the entire population. However, LAT was not an independent prognostic factor. Further subgroup analyses showed that LAT improved OS from 8.0 months to 10.0 months in patients with metastases other than bone/liver (HR = 0.759, 95% CI: 0.600–0.961, *p* = 0.022). LAT was not a prognostic factor in patients with bone/liver metastases (HR = 0.995, 95% CI: 0.799–1.239, *p* = 0.961). After PSM, the median OS was 8.0 months (95% CI: 7.2–8.8 months) and patients who received LAT had a better OS than patients without LAT (HR = 0.796, 95% CI: 0.653–0.968, *p* = 0.023). Patients with metastases other than bone/liver could benefit from LAT compared with those with bone/liver metastases.

**Conclusions:**

Our study indicated that metastatic ESCC patients with metastases other than bone/liver could derive additional benefit from LAT with systemic chemotherapy.

## Introduction

Esophageal cancer (EC) is the seventh most frequent cancer and had 544,076 estimated new cases of cancer deaths worldwide in 2020, according to the GLOBOCAN database (1). Esophageal squamous cell cancer (ESCC) accounts for more than 90% of EC in Asia and is closely associated with having hot food or water and alcohol consumption (2). About 20.0% of patients present with stage IV at the time of diagnosis (3). Chemotherapy was the standard treatment before the appearance of novel systemic therapy, such as immunotherapy and target therapies (4–6). However, response rates to chemotherapy alone ranged from 20% to 40%, and the median survival time was only approximately 8 months (7). So far, clinical trials have reported that immune checkpoint inhibitors, like programmed death ligand-1 (PD-L1) inhibitors or programmed death (PD-1) inhibitors, could prolong the median progression-free survival (PFS) time and even median overall survival (OS) time in advanced ESCC patients compared with chemotherapy (8–13).

However, local therapy is not a typical first-line treatment for metastatic ESCC patients. The common distant metastatic sites include lung, liver, bone, brain, adrenal glands, or distant lymph nodes (14). Many metastases are suitable for radiation, surgery, or other local therapies. Previous studies reported that local ablative therapy (LAT) to the primary tumor or metastatic sites could relieve the symptoms of obstructions, subsequent malnutrition, chronic bleeding, or pains in metastatic ESCC patients (12, 15, 16). We wonder if the addition of LAT to chemotherapy could improve the survival time of metastatic ESCC patients.

An observational cohort study used data from the National Cancer Database to assess the efficacy of radiotherapy in metastatic EC patients. In this study, 12,683 patients treated with chemotherapy were analyzed, and 3/4 of them were adenocarcinomas. Radiotherapy was performed directly at the primary tumor, and the results showed that definitive dose radiotherapy (≥50.4 Gy) improved median OS compared to chemotherapy alone [11.3 months vs. 8.3 months; hazard ratio (HR) = 0.72, 95% confidence interval (CI): 0.70–0.74, *p* < 0.001] (17). Another retrospective study investigated 461 stage IV ESCC patients with oligometastases (≤3 metastases). Among them, 265 patients were treated with chemotherapy alone, and 196 patients received concurrent chemoradiotherapy (CRT) for all metastases. Patients with concurrent CRT had a superior median PFS (8.7 months vs. 7.3 months, *p* = 0.002) and a trend toward better median OS (16.8 months vs. 14.8 months, *p* = 0.056) compared to those receiving chemotherapy alone (18). The latest retrospective study analyzed 126 advanced ESCC patients and found that CRT provided survival benefit to patients with distant metastasis. The CRT group had a greater median PFS (9.9 months vs. 4.0 months, *p* = 0.0032) and longer median OS (12.9 months vs. 9.3 months, *p* = 0.029) (19).

As for surgery, a retrospective investigation analyzed 96 stage IV EC patients treated with neoadjuvant chemotherapy followed by CRT, with or without surgery. Patients who had surgery had a more satisfying disease-free survival (DFS) (14.6 months vs. 5.9 months, *p* = 0.021) and a better median OS [NR (not reached) vs. 20 months, *p* = 0.001] (20). Meanwhile, another retrospective research included 34 advanced ESCC patients with concurrent CRT and reported that the addition of surgery improved median survival time (MST) from 5.0 months to 11.0 months (HR = 3.857, 95% CI: 1.142–13.024, *p* = 0.030) (21).

Hence, aggressive LAT added to palliative chemotherapy may improve prognosis in metastatic ESCC patients. However, previous studies are almost retrospective studies with a limited number of enrolled patients. Our study analyzed the large-scale population from the SEER database to clarify the potential benefit of LAT and identify other prognostic factors in metastatic ESCC. Patients who will most likely benefit were also uncertain. We further studied the difference in patients with different metastatic sites to identify the patients who benefit most from LAT. Results support clinicians to select the most appropriate treatment and recommend aggressive LAT to proper patients.

## Materials and Methods

### Patient Selection

SEER Stat software (SEER*Stat, v8.3.8) was used to search the data from the Surveillance, Epidemiology, and End Results (SEER) database of metastatic ESCC patients between 2010 and 2016. The inclusion criteria were as follows: (1) adults aged 18 years or older; (2) a pathological diagnosis of primary ESCC according to positive histology; (3) American Joint Committee on Cancer (AJCC) (7th Edition) TNM (tumor, node, metastasis) stage IV; (4) received chemotherapy; (5) complete chemotherapy, radiotherapy, and surgery information; and (6) a record of cancer-related death and OS. The following data were extracted: year of diagnosis, age, gender, race, AJCC (7th Edition) TNM stage, metastases at diagnosis, treatment (including chemotherapy, radiotherapy, and surgery), OS, and LAT (radiotherapy or surgery).

### Statistical Methods

SPSS 25.0 (SPSS Inc., USA) was used for statistical analysis. OS time was defined as the time of diagnosis to the date of death or last follow-up. The chi-square test was conducted to analyze the difference in baseline characteristics between every two groups. The Cox proportional hazard regression was used for univariate and multivariate analysis to identify potential prognostic factors. Factors with *p* < 0.05 in univariate analysis were included in the multivariate analysis. The estimated HR and 95% CI were calculated. Propensity score matching (PSM) was used to account for differences in patient characteristics among the two groups. The Kaplan–Meier method was used to create survival curves, calculate the median survival time, and compare prognosis between groups with the log-rank *p* test. *p*-values of <0.05 indicate statistical significance.

## Results

### Patient Characteristics

We identified 720 metastatic ESCC patients treated with chemotherapy. The baseline characteristics are listed in **Table 1**. Patients were diagnosed between 2010 and 2015. A total of 139 patients were diagnosed in 2010, 114 patients were diagnosed in 2011, 111 patients were diagnosed in 2012, 124 patients were diagnosed in 2013, 109 patients were diagnosed in 2014, and 123 patients were diagnosed in 2015. The median age at diagnosis of the entire population was 64 years (range: 39–93 years), and most patients (83.1%) were younger than 70 years old. Male was the main gender type (73.6%), and principal patients were white (59.9%). A total of 427 (59.3%) patients were T1–2, and 506 (70.3%) patients had positive lymph nodes. All patients were stage IV (M1) at the time of diagnosis. Lung metastases were the most common, followed by liver metastases and bone metastases (*n* = 277, 233, and 145, respectively). Of these, only 18 patients had brain metastases. Other metastases and the metastases numbers of each patient were not provided.

**Table 1:** The correlation between clinical parameters and LAT use.

		LAT (*n* = 455)	Non-LAT (*n* = 265)	*P*
Year of diagnosis				
2010	139 (19.3%)	92	47	0.148
2011	114 (15.8%)	75	39	
2012	111 (15.4%)	67	44	
2013	124 (17.3%)	67	57	
2014	109 (15.1%)	68	41	
2015	123 (17.1%)	86	37	
Age				
Median (range)	64 (39–93)	63 (39–93)	64 (39–91)	
<70	598 (83.1%)	378	220	0.984
≥70	122 (16.9%)	77	45	
Gender				
Male	530 (73.6%)	335	195	0.990
Female	190 (26.4%)	120	70	
Race				
White	431 (59.9%)	273	158	0.217
Black	201 (27.9%)	120	81	
Others	88 (12.2%)	62	26	
T				
T1–2	427 (59.3)	249	178	<0.001
T3–4	293 (40.7)	206	82	
N				
N0	214 (29.7%)	123	91	0.039
N+	506 (70.3%)	332	174	
Metastases at diagnosis				
Bone metastases	145 (20.1%)	96	49	0.400
No bone metastases	575 (79.9%)	359	216	
Brain metastases	18 (2.5%)	17	1	0.005
No brain metastases	702 (97.5%)	438	264	
Liver metastases	233 (32.4%)	104	129	<0.001
No liver metastases	487 (67.6%)	351	136	
Lung metastases	277 (38.5%)	179	98	0.530
No lung metastases	443 (61.5%)	276	167	

Of this population, 63.2% of patients (*n* = 455) received LAT, including radiotherapy (*n* = 444), primary site surgery (*n* = 12), or lymph node dissection (*n* = 27). There were no significant differences in the distributions of diagnosis year, age, gender, race, bone metastases, and lung metastases between the two groups (*p* > 0.05 for all). However, T stage (*p* < 0.001), N stage (*p* = 0.039), brain metastases (*p* = 0.005), and liver metastases (*p* < 0.001) were associated with LAT usage (**Table 1**). Thus, patients with T3–4, N+, brain metastases, and without liver metastases are more inclined to receive LAT.

### Univariate and Multivariate Analyses in Entire Population

**Figure 1:**
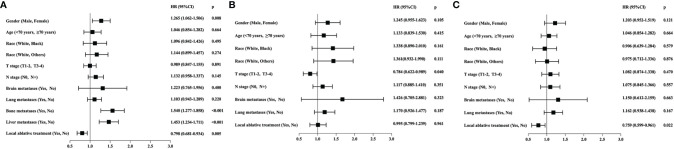
**(A)** Prognostic factors for overall survival (OS) through univariate analysis in all the enrolled metastatic ESCC patients from SEER database (*n* = 720). Gender (HR = 1.265, 95% CI: 1.062–1.506; *p* = 0.008), bone metastases (HR = 1.549, 95% CI: 1.277–1.858; *p* < 0.001), liver metastases (HR = 1.453, 95% CI: 1.234–1.711; *p* < 0.001), and local ablative treatment (LAT) (HR = 0.798, 95% CI: 0.681–0.934; *p* = 0.005) were associated with OS. **(B)** Prognostic factors for OS through univariate analysis in subgroup patients with bone/liver metastases (*n* = 336). T stage (HR = 0.784, 95% CI: 0.622–0.989, *p* = 0.040) was the only prognostic factor, and LAT was not associated with OS (HR = 0.995, 95% CI: 0.799–1.239, *p* = 0.961). **(C)** Prognostic factors for OS through univariate analysis in subgroup patients with metastases other than bone/liver (*n* = 384). LAT was a significant prognostic factor (HR = 0.759, 95% CI: 0.599–0.961, *p* = 0.022).

Results of univariate analysis in the entire population are shown in **Figure 1A**. Univariate analysis specified that gender (*p* = 0.008), bone metastases (*p* < 0.001), liver metastases (*p* < 0.001), and LAT (*p* = 0.005) were associated with OS in metastatic ESCC patients receiving chemotherapy. The multivariate analysis identified that gender (HR = 1.220, 95% CI: 1.024–1.453, *p* = 0.026), bone metastases (HR = 1.559, 95% CI: 1.292–1.882, *p* < 0.001), and liver metastases (HR = 1.457, 95% CI: 1.237–1.716, *p* < 0.001) were independent prognostic factors in the entire population. However, LAT was not an independent prognostic factor.

### Univariate and Multivariate Analyses in Patients With Different Metastatic Sites

To further clarify the role of LAT, we divided patients into two groups according to the existence of bone or liver metastases at diagnosis. A total of 336 patients had bone/liver metastases, and 384 patients had metastases other than bone/liver. The clinical characteristics are compared in **Table 2**.

**Table 2:** The clinical parameters between groups with bone/liver metastases or other metastases.

		With bone/liver metastases (*n* = 336)	With metastases other than bone/liver (*n* = 384)	*P*
Year of diagnosis				0.338
2010	139	53	86	
2011	114	58	56	
2012	111	55	56	
2013	124	61	63	
2014	109	50	59	
2015	123	59	64	
Age				
Median (range)	64 (39–93)	59 (39–91)	61 (41–93)	
<70	598	283	315	0.433
≥70	122	53	69	
Gender				
Male	530	262	268	0.013
Female	190	74	116	
Race				
White	431	208	223	0.179
Black	201	95	106	
Others	88	33	55	
T				
T1–2	427	223	204	<0.001
T3–4	293	113	180	
N				
N0	214	112	102	0.047
N+	506	224	282	
Metastases at diagnosis				
Brain metastases	18	8	10	0.848
No brain metastases	702	328	374	
Lung metastases	277	111	166	0.005
No lung metastases	443	225	218	
LAT	455	178	277	<0.001
Non-LAT	265	158	107	

There were no significant differences in the distributions of diagnosis year, race, and brain metastases between the two groups (*p* > 0.05 for all). Patients with bone/liver metastases were more likely to be male (*p* = 0.013), with T1–2 (*p* < 0.001), N0 (*p* = 0.047), without lung metastases (*p* = 0.005), and had less chance to receive LAT (*p* < 0.001) compared with patients with other metastases (**Table 2**).

Univariate analysis of subgroup with bone/liver metastases revealed that T stage (HR = 0.784, 95% CI: 0.622–0.989, *p* = 0.040) was the only prognostic factor, and LAT was not associated with OS (HR = 0.995, 95% CI: 0.799–1.239, *p* = 0.961) (**Figure 1B**). However, univariate analysis of the subgroup with metastases other than bone/liver metastases observed that LAT was a significant prognostic factor (HR = 0.759, 95% CI: 0.599–0.961, *p* = 0.022) (**Figure 1C**). The multivariate analysis further indicated that LAT improved OS in patients with metastases other than bone/liver metastases (HR = 0.759, 95% CI: 0.600–0.961, *p* = 0.022).

### Survival Outcomes in the Matched Patients

As age, gender, race, T stage, N stage, and metastatic site were important factors according to the multivariate analyses, we further made a PSM with these factors between the “LAT” group and the “non-LAT group”. After PSM, each group had 215 patients and the two groups were well balanced (*p* > 0.05 for all) (**Table 3**).

**Table 3:** The clinical parameters of matched LAT and non-LAT groups.

		LAT (*n* = 215)	Non-LAT (*n* = 215)	*P*
Age				
Median (range)	64 (39–91)	64 (41–88)	64 (39–91)	
<70	352	176	176	1.000
≥70	78	39	39	
Gender				
Male	328	166	162	0.650
Female	102	49	53	
Race				
White	272	139	133	0.335
Black	108	48	60	
Others	50	28	22	
T				
T1–2	308	157	151	0.521
T3–4	122	58	64	
N				
N0	126	64	62	0.832
N+	304	151	153	
Metastases at diagnosis				
Bone metastases	87	42	45	0.719
No bone metastases	343	173	170	
Brain metastases	2	1	1	1.000
No brain metastases	428	214	214	
Liver metastases	201	99	102	0.772
No liver metastases	229	116	113	
Lung metastases	151	73	78	0.613
No lung metastases	279	142	137	

**Figure 2:**
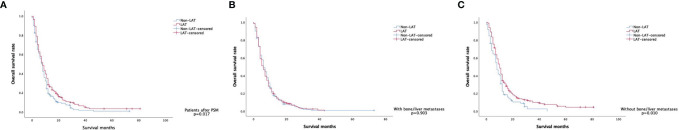
Kaplan–Meier curves of overall survival in **(A)** the matched patients (*n* = 430), **(B)** the matched patients with bone/liver metastases (*n* = 297), and **(C)** the matched patients with metastases other than bone/liver (*n* = 297).

The Kaplan–Meier survival curve showed that the median OS was 8.0 months (95% CI: 7.2–8.8 months) in all the patients after PSM. The OS of LAT and non-LAT groups had a significant difference [8.0 months (95% CI: 6.7–9.3 months) vs. 8.0 months (95% CI: 7.0–8.0 months), *p* = 0.017] (**Figure 2A**). Cox proportional hazard regression analysis found that patients who received LAT had a better OS than patients without LAT (HR = 0.796, 95% CI: 0.653–0.968, *p* = 0.023).

### Survival Outcomes in Patients With Different Metastases

To clarify the different role of LAT in patients with different metastatic sites, we further made a PSM according to age, gender, race, T stage, and N stage between the groups “with bone/liver metastases” and “with metastases other than bone/liver”. After PSM, data from 594 patients were available for analysis, and characteristics including age, gender, race, T stage, N stage, brain metastases, and lung metastases (*p* > 0.05 for all) were well balanced between the two groups (**Table 4**).

**Table 4:** The clinical parameters of matched groups with different metastases.

		With bone/liver metastases (*n* = 297)	With metastases other than bone/liver (*n* = 297)	*P*
Age				
Median (range)	64 (39–93)	63 (39–91)	65 (41–93)	
<70	500	251	249	0.822
≥70	94	46	48	
Gender				
Male	452	231	221	0.336
Female	142	66	76	
Race				
White	259	182	177	0.313
Black	161	84	77	
Others	74	31	43	
T				
T1–2	365	184	181	0.800
T3–4	229	113	116	
N				
N0	164	75	89	0.199
N+	430	222	208	
Metastases at diagnosis				
Brain metastases	15	6	9	0.433
No brain metastases	579	291	288	
Lung metastases	214	99	115	0.171
No lung metastases	380	198	182	

For the 297 patients with bone/liver metastases, the median OS was 6.0 months (95% CI: 5.1–6.9 months), and the LAT and non-LAT groups had no significant difference (*p* = 0.903) (**Figure 2B**). Patients with metastases other than bone/liver had a better median OS of 9.0 months (95% CI: 8.0–10.0 months), and patients with LAT improved median OS from 8.0 months to 10.0 months compared with non-LAT patients (*p* = 0.010) (**Figure 2C**). These results also supported the findings of our univariate and multivariate analyses.

## Discussion

Metastatic ESCC patients had a poor prognosis, and the 5-year survival rate was no more than 5% (7). LAT to the primary or metastatic sites may be suitable choices that not only relieve symptoms to improve life quality but also prolong the survival time in metastatic ESCC patients (18–21). However, previous studies were mostly retrospective studies with a limited number of patients. Up to now, conclusive results are lacking to affirm the advantages of LAT in metastatic ESCC patients.

Based on the large-scale population from the SEER database, our study calculated a median OS of 8.0 months in metastatic ESCC patients, and patients who received LAT had a superior OS to non-LAT patients (HR = 0.796, 95% CI: 0.653–0.968, *p* = 0.023). Compared with the largest previous study, the multicenter 3JECROG Survey, the median OS in our studies was much lower. The 3JECROG Survey summarized 3,977 ESCC patients who received chemotherapy and definitive radiotherapy at the primary tumor between 2002 and 2018 from nine institutions in China (3); 23.3% of patients (*n* = 928) were stage IV ESCC patients (according to the 6th TNM staging system), and the median OS of stage IVA and IVB patients was 17.2 months (95% CI: 15.0–19.3 months), and 16.6 months (95% CI: 14.7–18.5 months), respectively (3). No difference in OS was observed between stage IVA and stage IVB patients (*p* = 0.12) (3). Furthermore, the survival of patients who received concurrent CRT was better than that of patients who received sequential CRT (OS: 23.5 months vs. 17.6 months, *p* < 0.001) (3). Multivariate analysis in the concurrent CRT group found that patients receiving higher radiation dose (≥60 Gy) had a greater OS than those patients receiving low-dose radiotherapy (<50 Gy) (PFS: HR = 0.81, 95% CI: 0.68–0.98, *p* = 0.025; OS: HR = 0.77, 95% CI: 0.63–0.94, *p* = 0.009) (3).

Our study was different from the 3JECROG Survey. First, there were differences in the enrolled population: (1) We used the 7th TNM staging system instead of the 6th staging system in our study, and all the enrolled patients were M1. (2) Patients of the 3JECROG Survey were all Chinese and our study was based on an American database. Second, there were differences in multimodality treatment: (1) For the 3JECROG Survey, all patients received definitive radiotherapy at the primary site. However, radiation sites and doses were not provided in our study. Patients probably received radiotherapy for metastases or primary sites. (2) Some patients in our study received an operation of the primary site or lymph nodes, and the surgery may be very different from standard surgery. (3) Chemotherapy agents were heterogeneous in both studies and may affect the OS results.

The radiation dose of palliative intent for metastatic EC reportedly ranges from 30 to 50 Gy (21–23). However, a higher radiation dose with a definitive aim appears to produce better survival outcomes in metastatic EC patients. The impact of radiation dose was evaluated in another study consisting of 12,683 patients: 57% were treated with chemotherapy alone, 24% were treated with chemotherapy plus palliative dose radiotherapy, and 19% were treated with chemotherapy plus definitive dose radiotherapy (17). Radiotherapy was performed directed to the primary site, and the definitive dose of radiotherapy (≥50.4 Gy) improved median OS compared to those receiving chemotherapy alone (11.3 months vs. 8.3 months; HR = 0.72, 95% CI: 0.70–0.74, *p* < 0.001). However, palliative dose only slightly improved median OS from 8.3 months to 7.5 months (HR = 1.10, 95% CI: 1.07–1.13, *p* < 0.001) (17). The prognostic value of radiotherapy may be influenced by the radiation dose (definitive vs. palliative), sites (primary site vs. metastases; partial vs. all), and sequence (concurrent or sequential with chemotherapy), which need further randomized controlled clinical trials (RCTs) to answer this question.

The strength of our study is that we analyzed data from the SEER database, including a large number of metastatic ESCC patients, demonstrating continuous treatment and survival data for 6 years. LAT was applied in 63.2% of patients (*n* = 455), including radiotherapy (*n* = 444), primary site surgery (*n* = 12), or lymph node dissection (*n* = 27). It reveals the clinician’s choice of LAT for metastatic ESCC patients in the real world. Univariate and multivariate analyses of the entire population demonstrated that gender (HR = 1.220, 95% CI: 1.024–1.453, *p* = 0.026), bone metastases (HR = 1.559, 95% CI: 1.292–1.882, *p* < 0.001), and liver metastases (HR = 1.457, 95% CI: 1.237-1.716, *p* < 0.001) were independent prognostic factors.

Moreover, our study is the first to identify the effect of metastatic sites on the benefit of LAT in metastatic ESCC patients. LAT could improve median OS from 8.0 months to 10.0 months in patients with metastases other than bone/liver (HR = 0.759, 95% CI = 0.600–0.961, *p* = 0.022) and has no sense in patients with bone/liver metastases (*p* = 0.903). Another retrospective study of 198 stage IV ESCC patients reported that the CRT group had a longer median OS (14.0 months vs. 11.0 months, *p* = 0.007) than the chemotherapy group (74.5% versus 45.3%, *p* = 0.001). Multivariate analysis identified CRT (CRT vs. chemotherapy: HR = 0.626, 95% CI: 0.437–0.898, *p* = 0.013) and solitary metastasis (solitary vs. multiple metastasis: HR = 0.621, 95% CI: 0.426–0.905, *p* = 0.037) as independent factors for better OS in this study (24). The number of metastases may also be a prognostic factor, but it was not provided from the SEER database in our study. However, the different roles of LAT in ESCC patients with different metastatic sites had not been reported before. Based on our study, metastatic sites may help predict the survival time of patients and determine whether to use LAT or not.

Based on our study, LAT could improve OS in patients with metastases other than bone/liver. However, the prognosis of metastatic ESCC patients remains poor with LAT. Now, PD-1/PD-L1 inhibitors have emerged as a therapeutic option in advanced or metastatic patients. Previous studies reported that radiotherapy could enhance the anti-tumor immunity, break the resistance to immunotherapy, and induce a synergistic effect with PD-1/PD-L1 inhibitors in various cancers (25–27). The ATTRACTION-3 (8), KEYNOTE-181 (9), ESCORT (10), and ESCORT-1st (28) trials have led to remarkable changes in ESCC patients with the introduction of PD-1/PD-L1 inhibitors. So far, the combination of chemotherapy and pembrolizumab was approved as first-line treatment in metastatic ESCC patients by the National Comprehensive Cancer Network (NCCN). Meanwhile, pembrolizumab or nivolumab alone was preferred as second-line or subsequent therapy. However, very few studies evaluated the efficacy of combining radiotherapy with PD-1/PD-L1 inhibitors in metastatic EC patients. A phase Ib trial, NCT03222440, evaluated concurrent camrelizumab and radiotherapy (60 Gy/30 fr) as first-line therapy in 20 ESCC patients and observed two (11.1%) patients with complete response (CR), 13 (72.2%) with a partial response (PR), and three (16.7%) with a stable disease (SD) (29). More phase III RCTs are needed to further calculate the role of radiotherapy in immunotherapy.

It is worthy to note that our study had potential limitations. First, because of the deficiency of the SEER database, we were incapable of obtaining detailed data, especially the specifics on treatment (chemotherapy regimens, surgery progress, radiation site, dose and sequence, and the time of using LAT). Second, bias was inevitable because the SEER database does not mention possible prognostic factors, such as patient performance status, alcohol drinking history, smoking history, blood inflammatory factors, associated gene expression, and prior treatments. Finally, another limitation of this study is that our findings are not for those with adenocarcinomas or those with early-stage and locally advanced ESCC patients.

In conclusion, our study suggests that male, metastatic ESCC patients with bone/liver metastases may have poorer survival outcomes, and patients with metastases other than bone/liver could derive additional benefits from LAT with systemic chemotherapy. Our study support aggressive LAT in metastatic ESCC patients with metastases other than bone/liver. Due to the lack of convincing results, we recommend aggressive LAT usage be further tested in large-scale RCTs to define patients who will most likely benefit and evaluate the treatment-associated adverse events.

## Data Availability Statement

The original contributions presented in the study are included in the article/supplementary material. Further inquiries can be directed to the corresponding author.

## Ethics Statement

The studies involving human participants were reviewed and approved by the Ethics Committee of the Affiliated Cancer Hospital of Zhengzhou University. Written informed consent for participation was not required for this study in accordance with the national legislation and the institutional requirements.

## Author Contributions

HY and LY designed the study. HG improved study design and supervised this study with HY and LY. HY, KW, YL, and SL collected the data and drafted the manuscript. HY, KW and HG performed the statistical analysis. HG reviewed and edited the manuscript. All authors contributed to the article and approved the submitted version.

## Funding

This work was supported by the Science and Technology Department, Henan Province (grant numbers: SB201901113 and 192102310048).

## Conflict of Interest

The authors declare that the research was conducted in the absence of any commercial or financial relationships that could be construed as a potential conflict of interest.

The reviewer FM declared a shared affiliation with the authors to the Handling Editor at the time of the review.

## Publisher’s Note

All claims expressed in this article are solely those of the authors and do not necessarily represent those of their affiliated organizations, or those of the publisher, the editors and the reviewers. Any product that may be evaluated in this article, or claim that may be made by its manufacturer, is not guaranteed or endorsed by the publisher.
